# Analysis of coping capacities and cognitive biases of novice drivers—A questionnaire-based study

**DOI:** 10.1371/journal.pone.0297763

**Published:** 2024-02-16

**Authors:** Wang Xiang, Yonghe Zhang, Xin Pan, Xuemei Liu, Guiqiu Xu

**Affiliations:** 1 Hunan Key Laboratory of Smart Roadway and Cooperative Vehicle-Infrastructure Systems, Changsha University of Science and Technology, Changsha, Hunan, China; 2 State Grid Hunan Electric Power company Limited Economic & Technical Research Institute, Changsha, Hunan, China; 3 Hunan Key Laboratory of Energy Internet Supply-Demand and Operation, Changsha, Hunan, China; 4 Hunan Institute of Traffic Engineering, School of Traffic & Transportation Engineering, Hengyang, Hunan, China; 5 GuangDong Architectural Design & Research Institute Company Limited, Guangzhou, Guangdong, China; University of Valencia: Universitat de Valencia, SPAIN

## Abstract

Coping capacity is a key aspect of driver-vehicle interaction when drivers observe and make decisions, and is of great importance for drivers. However, different drivers have different self-cognition and assess their driving abilities differently, especially for novice drivers. Based on questionnaire data, this study has investigated the coping capacities of drivers in both static environments and dynamic environments. With the ANOVA analysis method and the structural equation model (SEM), this study has verified the effects of gender and driving factors (driving years, driving frequency, driving time) on drivers’ coping capacities based on drivers’ self-assessment scores and mutual assessment scores. Drivers’ self-assessment scores show significant effects of all factors on drivers’ coping capacities, and drivers’ mutual assessment scores show significant effects of all factors, excluding driving time, on drivers’ coping capacities. Also, it has been found that all drivers in the driving year group have cognitive biases. It seems that first-year drivers are always overconfident with their driving skills, while drivers with a driving experience of more than three years usually score driving skills of themselves and other drivers most conservatively. With increased exposure to various traffic conditions, experienced drivers are more aware of their limitations in dealing with complex traffic situations, while novice drivers do not know their lack of capability to properly respond to any unexpected situation they could encounter.

## Introduction

In the last decade, the number of novice drivers in China has increased rapidly. It is estimated that in 2021, the number of novice drivers (who have owned a driving license for less than three years [1]) in China was 79 million, accounting for 16% of the total number of all drivers in China in that year [2–4]. In motorized countries, novice drivers are less experienced drivers who are more likely to be involved in a traffic accident than experienced drivers [5–7].

Driving a vehicle is a complex and dynamic activity, which primarily requires three capabilities, which are the capabilities of perceiving, information processing, and vehicle manipulating [8]. In some literature, driving skill refers to the ability to perceive risk and manipulate a vehicle [9]. Controlling a vehicle (coping capacity) is the last step, and all the information obtained through vision and hearing and then processed by the brain should be used in controlling the vehicle.

The coordination between a driver’s perception and coping capacities may play a crucial role in that driver’s behavior and safety [10,11]. According to the Driving as an Everyday Competence Model and the Multi-factor Model for Enabling Driving Safety [12,13], those drivers who are accurately aware of their driving abilities are more likely to adopt appropriate driving strategies or tactics to regulate their own behaviors.

Overestimation of skill, also called the optimism bias [14,15], has been measured in a questionnaire with a comparison of drivers’ skills with an “average” driver’s skill. The results show that all drivers believe they are better than the "average driver" [16,17]. In particular, novice drivers, who usually misjudge their own driving skills, are more likely to overestimate their abilities than experienced drivers [6]. Previous studies have shown that drivers overestimating their driving skills could have a higher risk of exposing to an accident and a lower motivation in regulating their self-behaviors [18], while drivers underestimating their driving skills could over-constrain their driving behaviors, reduce their driving frequency or even stop driving [19].

As a daily activity, driving is affected by a variety of stressors [20,21]. Driver stress occurs primarily when individuals perceive their driving ability to be insufficient to manage the demands and hazards of driving [20,22]. Driver stress is transactional in nature, eliciting qualitatively different patterns of stress response, coping strategies, and driving performance [22–25]. In emergency situations, time constraints may result in perceptions that internal resources are insufficient to accomplish tasks effectively [26,27]. In complex driving situations, stress can reduce the driving ability of drivers who lack confidence [28–30].

It is essential for drivers to be accurately aware of their own driving skills to achieve driving safety [31]. Therefore, when driving a vehicle, they can take adequate precautions to secure their safety [32]. So, it is important to eliminate cognitive biases or at least reduce cognitive biases to some extent. This paper is to investigate the cognitive biases (overconfident, normal, or less confident) and coping capacities of novice drivers in both dynamic environments (for example, a car ahead suddenly brakes, or someone suddenly rushes out from the right side) and static environments (for example, the driver needs to pass through a narrow road, or a running vehicle suddenly skids in a rainy day). Factors such as gender and driving characteristics (driving year, driving frequency, and driving time) of drivers assessing the coping capacities of themselves and others have been taken into consideration in this study. Also, a structural equation model was constructed in this study to investigate the causal relationships between each factor and the self-assessment and mutual assessment results of drivers.

## Literature review

### Novice driver

Drivers with less than 3 years of driving experience are considered as novice drivers [1]. It showed that the number of accidents caused by new drivers with less than 3 years of driving experience accounts for more than 40% of the total number of driver accidents, and the number of deaths caused by accidents caused by new drivers accounts for nearly half of the total number of accidents caused by new drivers [33]. Paulius M. et al. pointed out that most accidents are caused by people who lack driving experience or do not have a driver’s license. The most common causes of road traffic accidents are drivers with 2–3 years of driving experience and 11–12 years of driving experience [34].

Laapotti explained this high percentage phenomenon with three main factors: exposure, driving style, and driving skills [35]. First, novice drivers have fewer exposures, fewer miles driven, and fewer encounters with a variety of situations than experienced drivers. Second, novice drivers take more risks than experienced drivers [36]. Finally, their driving skills are associated with less practice [37]. Novice drivers pose a greater threat to traffic safety due to their idiosyncrasies, so it is important to study novice drivers to reduce traffic accidents and improve road safety.

In order to reduce the accident rate of novice drivers, scholars have conducted related research in various aspects. By analyzing data from the Driver Behavior Questionnaire, numerous studies found the importance of early intervention in improving road safety, with the critical period being the initial few months [38–40]. Ellen M.M. et al. investigated the cognitive mechanisms of risky driving in novice drivers through a driving simulator and concluded that lower cognitive control and rewarding abilities predict risky driving and form the basis of their cognition [41]. Mark S.H. evaluated a hazard perception training course to minimize forgetfulness. It showed that training courses improve the skills of novice drivers in relation to crash risk, and that the longer the training period, the better the results obtained [42].

Many efforts have also been made in various countries to reduce the road safety hazards posed by novice drivers. In some European countries, driver training programs are divided into two phases, including the pre-licensing and post-licensing phases, and long-term (continuous) road safety education initiatives have also emerged [43]. In the United States, almost all states have adopted Graduated Driver Licensing (GDL) policies aimed at reducing the crash risk of novice teenage drivers by requiring extended periods of supervised practice and limiting drivers’ exposure to high-risk conditions [44].

### Coping capacity

Driving a vehicle requires a driver to perform a range of basic driving tasks, including lower-order driving competencies such as vehicle control, as well as higher-order driving competencies such as risk recognition and hazard perception [45,46]. Higher-order driving competencies are so important to drivers that assessment of hazard perception skills has become part of the graduated driver licensing system in some countries [47–49]. Although higher-order driving competencies are important, lower-order driving competencies like coping capacities should not be overlooked. Low-order driving competency is directly related to unifying information processed by the brain into vehicle control [50], and young drivers with poorer low-order driving ability are more likely to engage in risky driving behaviors [51].

Most research methods on coping capacity have focused on literature review, questionnaires and driving simulation experiments. Elizabeth A. et al. collected and organized the literature on young drivers’ driving ability and analyzed the roles of different driving ability structures and sub-processes [52]. Kong conducted a questionnaire survey and driving simulation experiment to study the cognitive bias of coping ability of young student drivers [53]. The driving simulator-based simulation experiment method can study the driving ability of drivers while they are maneuvering the vehicle, making the results more objective and realistic [54].

### Cognitive bias

To understand cognitive biases, it is important to understand the reasoning processes and methods of how drivers make decisions. Kahneman proposed two systems of human thought processing: the fast intuitive thinking system1 (“Type 1 thinking”) and the slow rational thinking system2 (“Type 2 thinking”) [55], and argued that heuristic thinking belongs to system 1 and allows people to reach conclusions faster [55,56].

Heuristic thinking may fail due to errors caused by incorrect reasoning and judgments, which are known as cognitive biases [57], and these cognitive biases can lead to perceptual and interpretive errors [56,58]. On the other hand, System 2 is more likely to arrive at correct judgments by adopting a more systematic, analytical and thoughtful approach to decision-making [55,56,59,60]. Based on the concept of dual process theory, both system 1 and system 2 thinking are necessary to make the correct judgment [60].

However, since the situations encountered by drivers while driving on the road are more urgent and do not allow for a long period of thinking by the driver, the driver’s judgments are usually based on heuristics, which are defined as the ability of the human brain to take shortcuts in solving problems and making quick judgments by drawing on past experiences [57,60,61].

Driving may seem automatic, however it is one of the most cognitively complex and potentially dangerous tasks people undertake on a daily basis [62]. Jongena revealed the cognitive mechanisms underlying risky driving in young novice drivers through a driving simulation experiment and verified that lower cognitive control constitutes its cognitive foundation [41]. Fu proposed a novel framework for predicting drivers’ cognitive behavior through driver actions based on a neural regression model from the perspective of eliminating cognitive bias due to age [63].

### Driver questionnaires and analytical methods

Driver self-administered questionnaires, as an effective method in driver characterization research, have been widely used in the collection of driver subjective characteristic data [11,64,65]. Especially in the fields of traffic accident analysis and driving psychology, it is often necessary to analyze and predict driver behavior through the subjective assessment of drivers’ own style and driving habits [66].

The methods of traffic questionnaires based on self-report include: the Manchester Driving Behavior Questionnaire (DBQ), Driving Behavior Inventory (DBI), and Driving Style Questionnaire (DSQ). Questionnaire (DSQ) [67]. Among the various forms and contents of questionnaires, the Manchester Driver Behavior Questionnaire constructed by Reason et al. [68] is one of the more commonly used standardized questionnaires, many scholars have conducted studies based on this questionnaire for the driver population in their own countries [69–71].

The analysis methods used for the questionnaire are mainly the following four categories, exploratory factor analysis and confirmatory factor analysis, correlation analysis and regression analysis, dominance analysis and path analysis, analysis of variance and analysis of covariance [72]. Li et al. used ANOVA to find that risk vigilance awareness was higher among older and long driving age drivers than younger and shorter driving age drivers in the trucking industry [73]. Xiang et al. [74], and Liu et al. [75] analyzed the cognitive bias analysis of perceived competence of drivers of different driving ages by using ANOVA and structural equation modeling (SEM) methods on questionnaire data.

### Aim and hypotheses of the study

The aim of this study is to investigate the cognitive bias in the coping capacity of drivers of different driving years under dynamic environments (in which there are influences coming from other vehicles or pedestrians) and static environments (in which the driver is not affected by other vehicles or pedestrians) based on the data from the questionnaire survey. Based on the literature available so far, we hypothesized that (i) driving year presents a positive correlation with self-assessment of coping capacity and a positive correlation with the mutual assessment of coping capacity. (ii) driving year presents a negative correlation with cognitive bias in coping capacity.

## Method

### Participants

In this study, a total of 1031 questionnaires were collected through the online questionnaire platform, and 319 valid questionnaires were obtained after excluding 712 invalid questionnaires that were blank, incompletely filled out, consecutively choosing the same answer, or having obvious patterns or errors in the answers. Table 1 shows the statistical data on the characteristics of all correspondents who have submitted a valid questionnaire in this study.

**Table 1:** Demographic information of participants.

Personal information		Number of participants	Accounting for /%	Driving information		Number of participants	Accounting for /%
Gender	Male(M)	177	55.49	Driving Years	< 1	93	29.15
	Female(F)	142	44.51		1 ~ 3	131	41.07
					> 3	95	29.78
Age	18~25	216	67.71	Driving frequency (per week)	0~2	200	62.69
	26~40	68	21.32		3~ 5	45	14.11
	>40	35	10.97		>5	74	23.20
Education	High school(H)	47	14.73	Driving time (minutes)	< 30	87	27.27
	Bachelor(B)	236	73.98		30 ~ 60	156	48.90
	Master(Ma)	36	11.29		> 60	76	23.83
Major	Natural science(N)	153	47.96				
	Liberal arts(L)	102	31.97				
	Other majors(O)	27	8.46				

### Methodology

In this study, an anonymous questionnaire survey approved in writing by the Ethics Committee of Changsha University of Science & Technology was conducted without additional participant consent required. All authors had no access to information that could identify individual participants during or after data collection. Dr. Wang Xiang has drafted this questionnaire on drivers, participants have been recruited to the study in September 2020, and the data of the questionnaire has been analyzed. Dr. Wang Xiang and Yonghe Zhang from the Transportation Research Center administered the survey. Also, the details of the questionnaire can be found in the S1 Appendix.

#### Questionnaire

**Fig 1:**
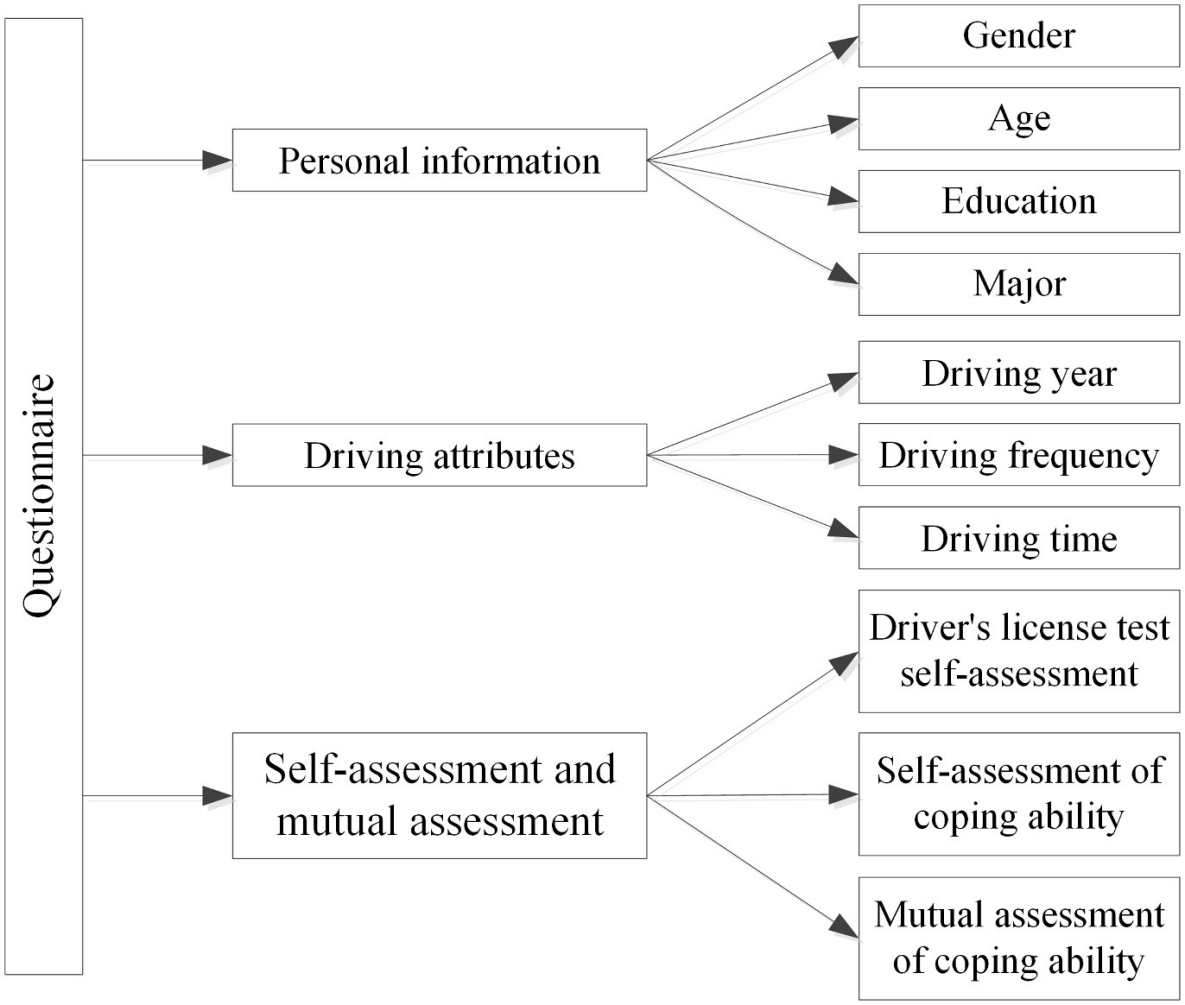
Framework of questionnaire design.

The questionnaire consists of three sections, namely driver’s personal information, driver’s driving attributes, and driver’s rating scores (as shown in Fig 1). In the first two sections, drivers’ personal information includes such information as gender, age, education, and major, and driving attributes include the information of driving year, driving frequency, and driving time. In the driver rating section, participants first need to provide a score on their own performance in the driving test, and then they need to evaluate their own coping capacities and the coping capacities of other drivers. To evaluate their own coping capacities, the correspondents need to score their performance under both dynamic conditions (as described in the Introduction section of this paper) and static conditions (as described in the Introduction section of this paper). Also, to evaluate the coping capacities of other drivers (mutual evaluation), the participants need to score the performance of other drives in both dynamic environments (in which there are influences coming from other vehicles or pedestrians) and static environments (in which the driver is not affected by other vehicles or pedestrians). In this section, the performance of drivers is scored on a scale of 1 to 10, with 1 representing the weakest ability and 10 representing the highest ability.

Drivers’ mean self-assessment scores (SAS) and mean mutual assessment scores (MAS) under different genders, driving years, driving frequency, and driving time are shown in Tables 2 and 3, respectively.

**Table 2:** Descriptive statistics of mean self-assessment scores.

Variable		Dynamic situations (With human or car)			Static situations (With no human or car)		
		Within 1 year	1 to 3 years	Over 3 years	Within 1 year	1 to 3 years	Over 3 years
Gender	Male	7.11	7.58	8.04	7.47	7.75	8.22
	Female	6.41	6.56	6.83	6.63	6.85	6.78
Driving years		6.76	7.15	7.52	7.05	7.37	7.6
Driving frequency (per week)	0 to 2 times	6.57	6.84	6.47	6.83	7.13	6.37
	3 to 5 times	7	7.44	7.74	7.4	7.56	8.05
	Over 5 times	7.62	8.22	8.45	8	8.22	8.61
Driving time (minutes)	Less than 30	6.43	6.67	6.24	6.57	6.5	5.88
	30 to 60	6.84	7.24	7.68	6.86	7.62	7.84
	More than 60	7.05	7.73	8	8.05	8.19	8.21

**Table 3:** Descriptive statistics of mean mutual assessment scores.

Variable		Driving years		
		Within 1 year	1 to 3 years	Over 3 years
Gender	Male	5.93	7.35	8.69
	Female	5.72	7.05	8.33
Driving years	Within 1 year	6.24	7.55	8.85
	1 to 3 years	5.9	7.27	8.63
	Over 3 years	5.35	6.81	8.08
Driving frequency (per week)	1 to 2 times	5.77	7.09	8.43
	3 to 5 times	5.84	7.31	8.47
	Over 5 times	6	7.5	8.85
Driving time (minutes)	Less than 30	5.89	7.23	8.4
	30 to 60	5.85	7.24	8.56
	More than 60	5.75	7.14	8.63

#### Validity test and reliability test

Firstly, the SPSS26.0 software was used to test the applicability of participants’ scores. The test yielded a significant value of Bartlett’s test of sphericity p, which is lower than 0.001, and a Kaiser-Meyer-Olkin (KMO) value of 0.776, indicating that the assumptions made in the factor analysis all hold.

Secondly, a Principal Component Analysis was performed on the collected survey data, with the results shown in Tables 2 and 3. The cumulative variance contribution rate of the three extracted principal components shown in Table 4 is 71.131%, indicating that they can fully reflect the original data.

**Table 4:** Table of relevant parameters of each factor.

Total Variance Explained									
Component	Initial Eigenvalues			Extraction Sums of Squared Loadings			Rotation Sums of Squared Loadings		
	Total	% of Variance	Cumulative %	Total	% of Variance	Cumulative %	Total	% of Variance	Cumulative %
1	4.760	43.277	43.277	4.760	43.277	43.277	3.514	31.948	31.948
2	1.957	17.788	61.065	1.957	17.788	61.065	2.286	20.783	52.731
3	1.107	10.066	71.131	1.107	10.066	71.131	2.024	18.400	71.131

Then, the reliability of the questionnaire was tested, with the results shown in Table 5. It can be seen that the obtained values of Cronbach’s α and Composite Reliability (CR) are all greater than 0.8, and the obtained value of Average Variance Extracted (AVE) is greater than 0.5, indicating that the questionnaire has good internal consistency and convergent validity.

**Table 5:** Table of relevant parameters of each factor.

Component index NO.	Latent Variable	Observed Variable	Factor Loadings	Cronbach’s α	CR	AVE
1	Driving license test	Reverse warehousing	0.776	0.869	0.8843	0.5617
		Parallel parking	0.771			
		S-type driving	0.828			
		Half-slope start	0.743			
		Road test	0.729			
		Theoretical exam	0.636			
2	Mutual assessment	Mutual-within 1 year	0.831	0.806	0.8738	0.6997
		Mutual-1 to 3years	0.935			
		Mutual-over 3 years	0.731			
3	Self-assessment	Self-dynamic	0.908	0.955	0.9076	0.8308
		Self-static	0.915			

#### One-way analysis of variance

Based on survey data, a method of variance analysis (ANOVA) was used to test the effects of drivers’ personal information, driving attributes, and driving license tests on their coping capacities, which were further compared when there were significant differences among these effects (Refer to Tables 6 and 7).

**Table 6:** Results of One-way ANOVA analysis of the relationships between drivers’ SAS and drivers’ driving characteristics.

Variables			Gender	Driving years	Driving frequency	Driving time
Self	Dynamic	<1	18.284**	8.031**	10.437**	3.738*
		1~3	24.952**		14.054**	6.637**
		>3	14.388**		19.367**	7.554**
	Static	<1	24.568**	4.026*	12.221**	28.506**
		1~3	21.560**		9.167**	27.191**
		>3	17.852**		22.885**	12.334**

**At 0.01 significance level.

*At 0.05 significance level.

**Table 7:** Results of One-way ANOVA analysis of the relationships between drivers’ MAS and drivers’ driving characteristics.

Variables		Gender	Driving year	Driving frequency	Driving time
Mutual	<1	1.734	10.216**	0.723	0.198
	1~3	4.784*	9.175**	3.207*	0.172
	>3	7.145**	10.604**	3.347*	0.778

**At 0.01 significance level.

*At 0.05 significance level.

#### Structural equation modeling

Structural equation modeling (SEM) was performed to explore the direct and indirect causal relationships between each factor and the drivers’ coping capacities. SEM can weigh the influences of exogenous and endogenous variables. Combining SEM with factor analysis and simultaneous equation model has been widely used as a linear-in-parameter multivariate statistical modeling technique. In this study, SEM has been used to explore the causal relationships between driving factors such as driving years, driving frequency, and driving time with drivers’ self-assessment and mutual-assessment scores so as to reveal the cognitive biases of drivers. The IBM SPSS Analysis of Moment Structures (AMOS 24) software package was used to perform the SEM analysis.

## Results

### Self-assessment

**Fig 2:**
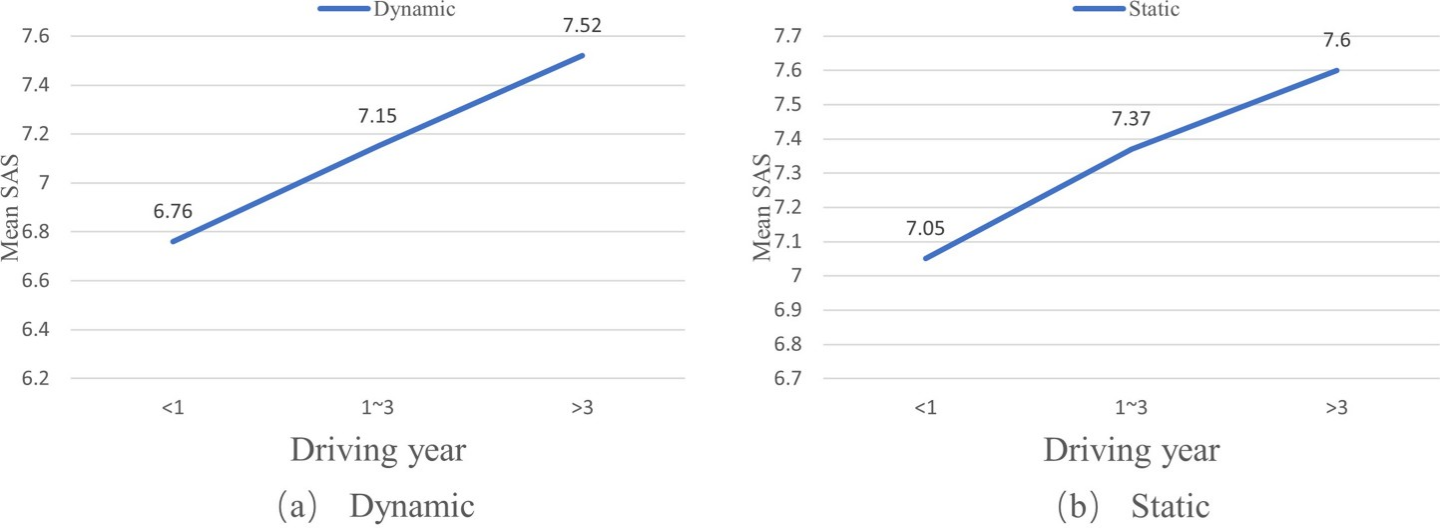
Effects of driving years on self-assessment scores (SAS) of drivers in different driving year groups.

A one-way ANOVA analysis was conducted to explore the relationship between the driving year factor and drivers’ self-assessment scores on their coping capacities under both dynamic and static conditions. The analysis showed significant effects of the driving year factor on drivers’ SAS under both dynamic (F = 8.031, p < 0.01) and static conditions (F = 4.026, p < 0.05). Generally, drivers’ SAS on their coping capacities increase with their driving years, as shown in Fig 2.

**Fig 3:**
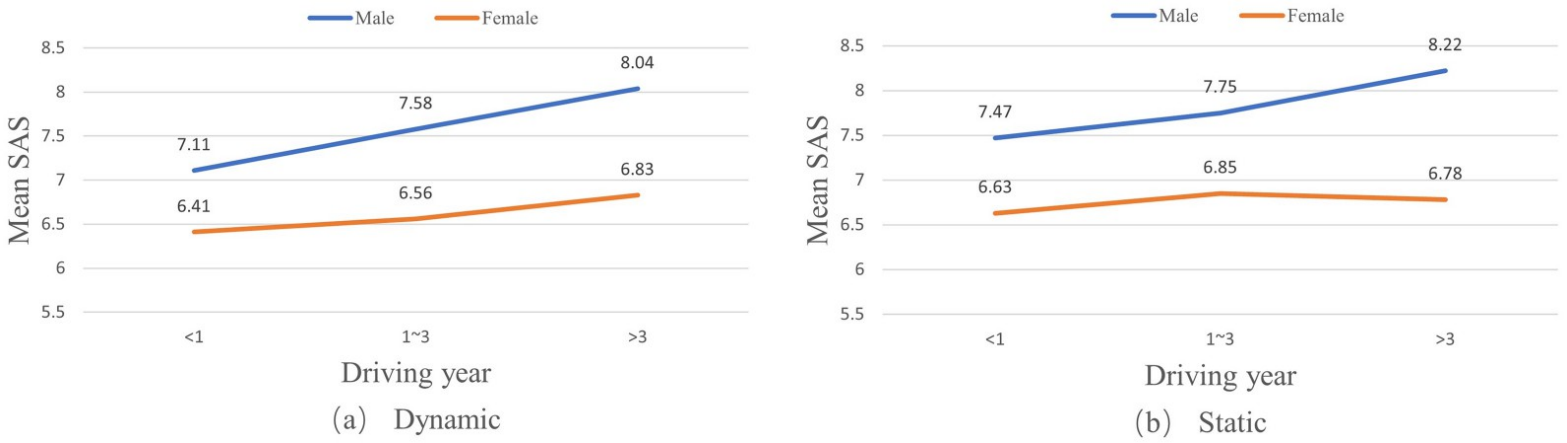
Effects of gender on self-assessment scores (SAS) of drivers in different driving year groups.

Moreover, this study has investigated the effects of drivers’ gender, driving frequency, and driving time on SAS. Male drivers’ SAS are higher than female drivers’ SAS for both dynamic and static conditions (see Fig 3). Also, the SAS of all male drivers and female drivers with driving experience of no more than three years increase with their driving years.

**Fig 4:**
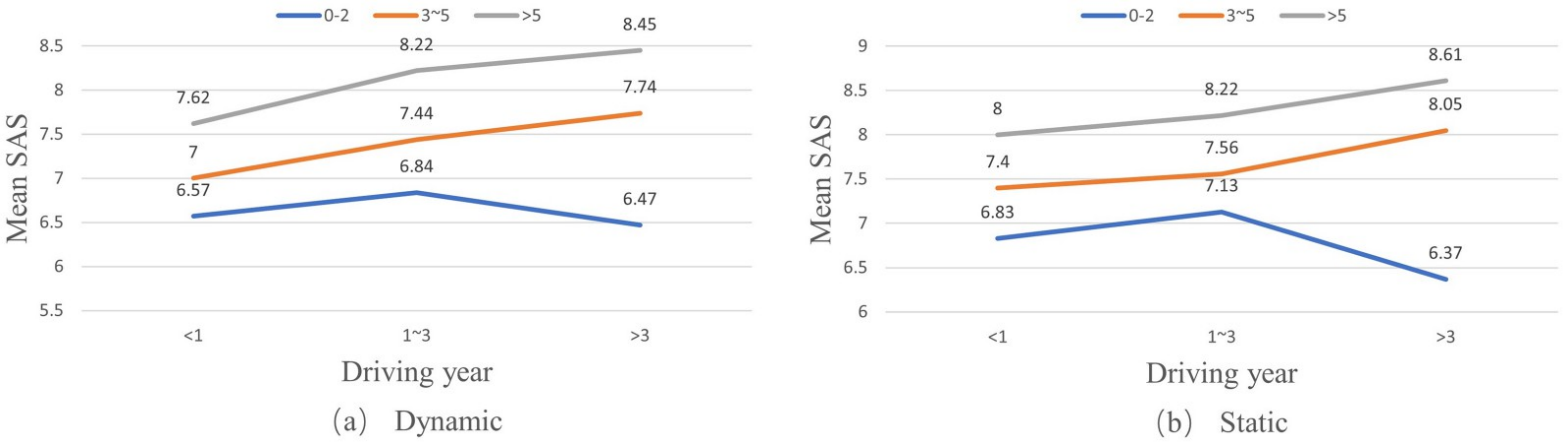
Effects of driving frequency on self-assessment scores (SAS) of drivers in different driving year groups.

**Fig 5:**
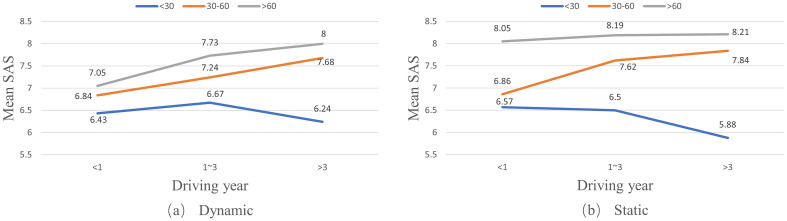
Effects of driving time on self-assessment scores (SAS) of drivers in different driving year groups.

Similarly, there are significant effects of driving frequency and driving time on drivers’ SAS under both static and dynamic conditions (as shown in Table 6). Also, drivers’ SAS on their coping capacities increase with their driving experience; that is, a higher driving frequency and more driving time (as shown in Figs 4 and 5, respectively).

### Mutual assessment

This study has also analyzed the relationships between the characteristics of drivers and their MAS. As shown in Table 5, drivers’ genders, ages, driving years, and driving frequency all have a significant effect on their MAS, while drivers’ education, majors, and driving time have no significant impact on drivers’ MAS.

**Fig 6:**
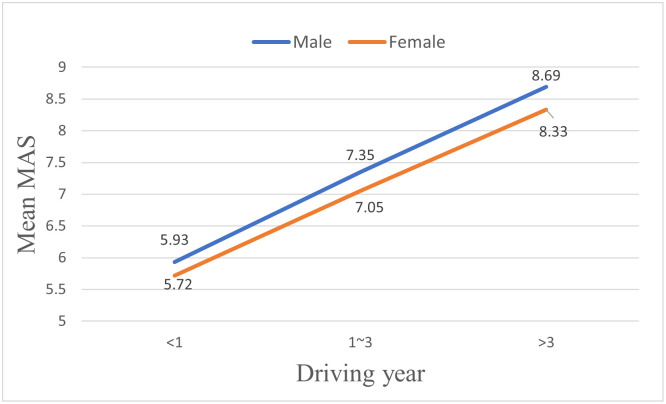
Effects of gender on mutual assessment scores (MAS) of drivers in different driving year groups.

**Fig 7:**
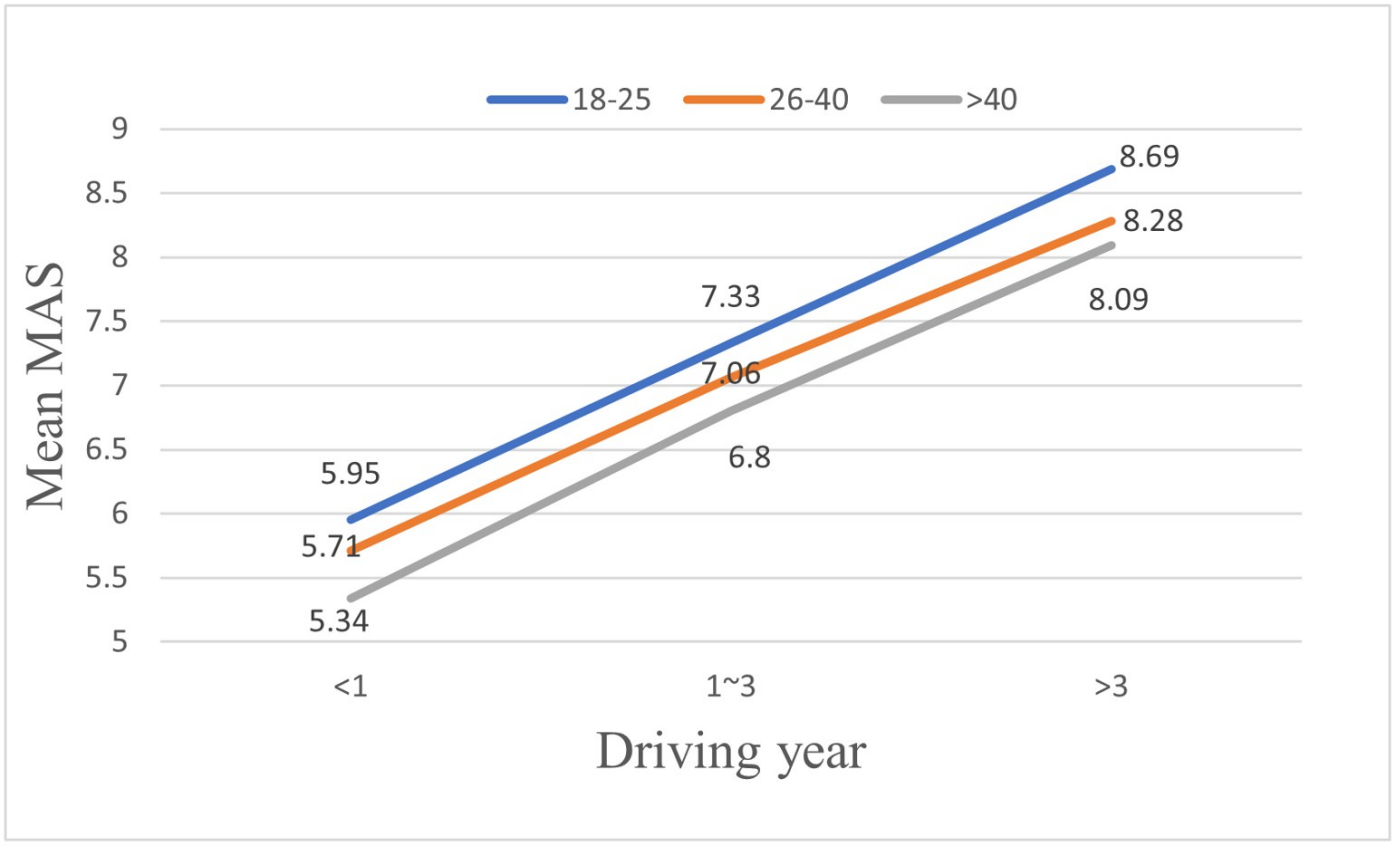
Effects of age on mutual assessment scores (MAS) of drivers in different driving year groups.

**Fig 8:**
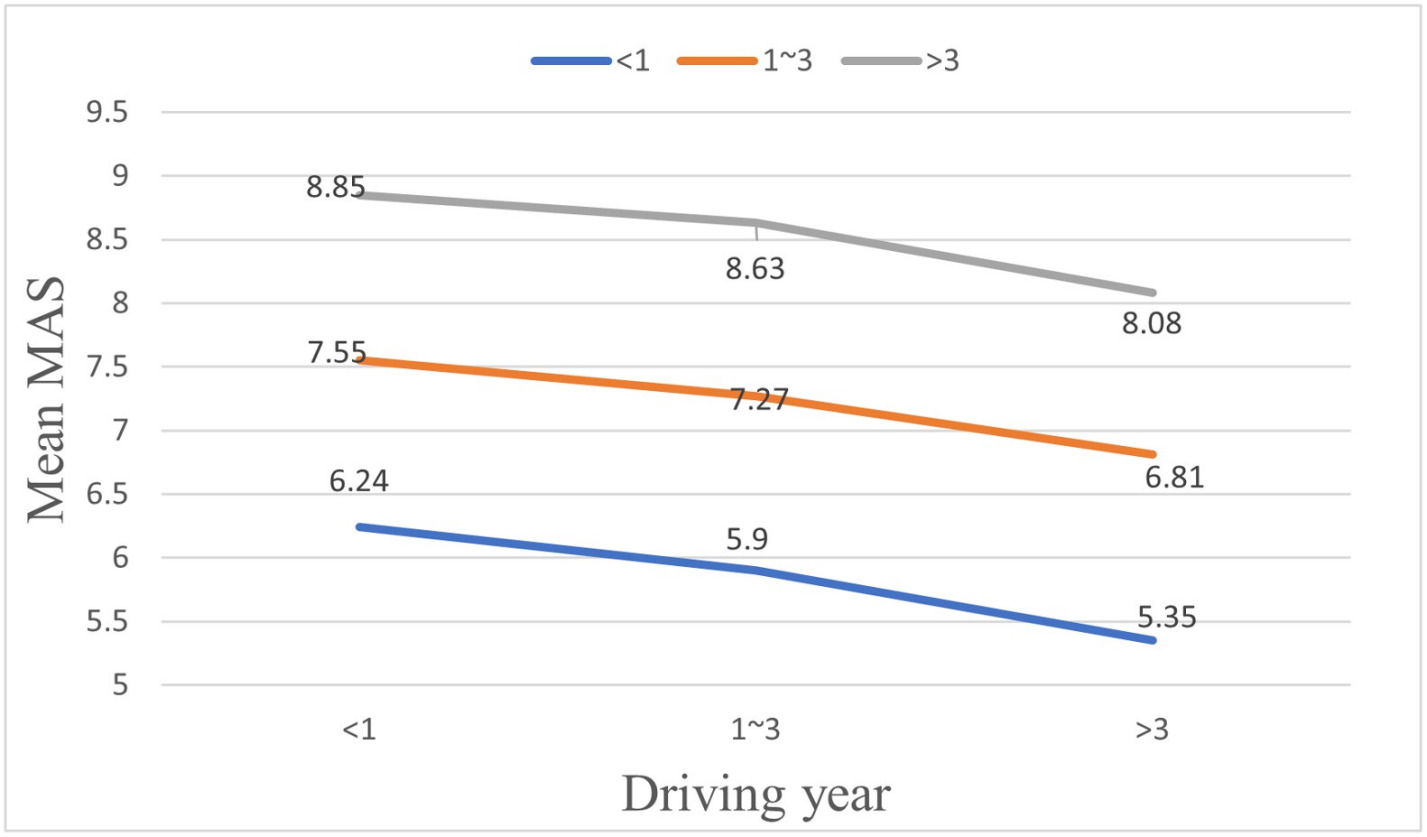
Effects of driving years on mutual assessment scores (MAS) of drivers in different driving year groups.

**Fig 9:**
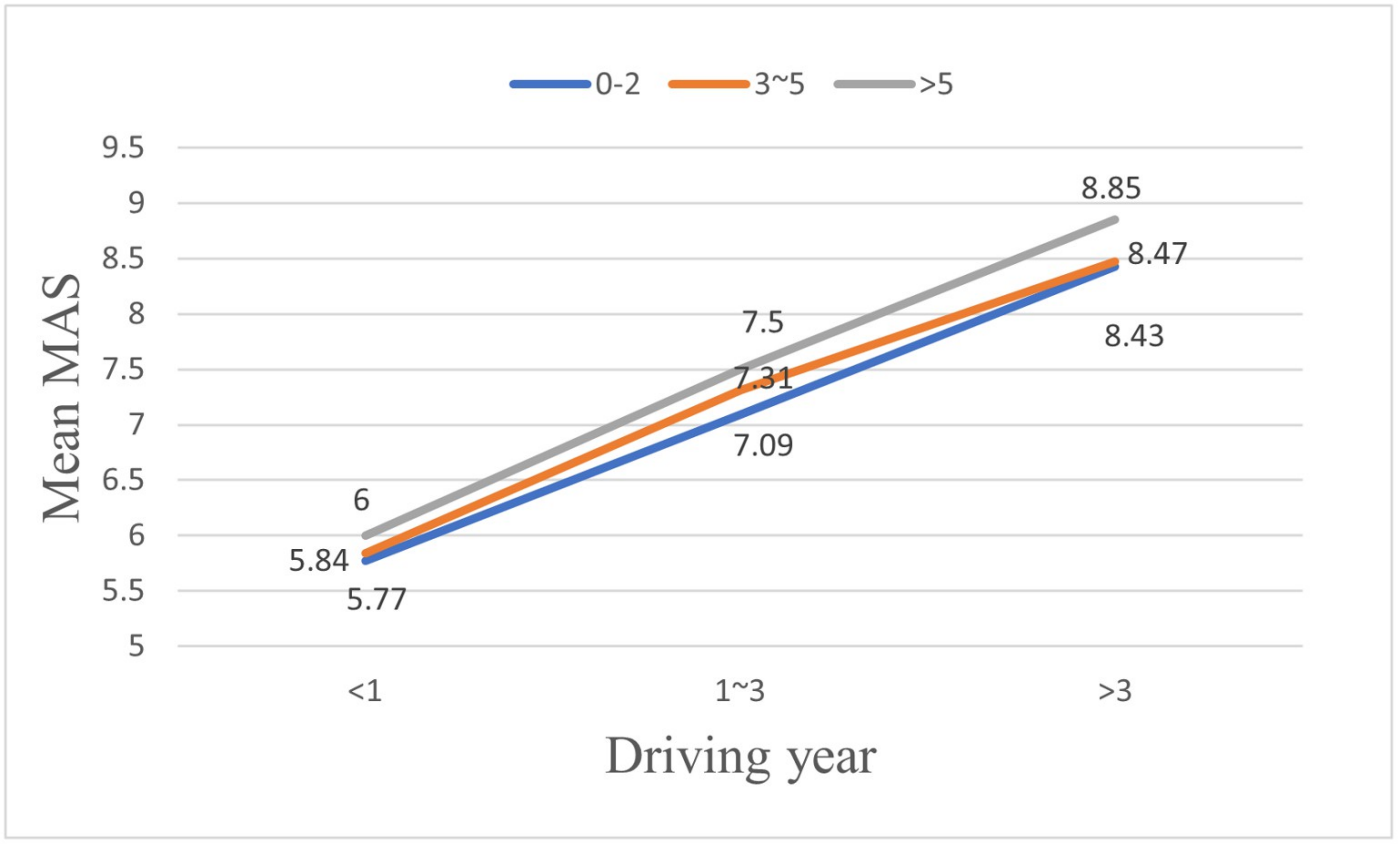
Effects of driving frequency on mutual assessment scores (MAS) of drivers in different driving year groups.

In terms of gender, as shown in Fig 6, the mutual assessment scores (MAS) provided by male drivers on their coping capacities are significantly higher than those scores provided by female drivers. In terms of age, as shown in Fig 7, the older the drivers are, the lower MAS they would provide. Similar to the results shown in the previous section on drivers’ SAS, drivers’ MAS increase significantly with their driving years and driving frequency, as shown in Figs 8 and 9. However, there is no significant effect of gender (F = 1.734, p > 0.05) or driving frequency (F = 0.723, p > 0.05) on MAS provided by drivers with driving experience lower than one year.

### Structural equation model

**Fig 10:**
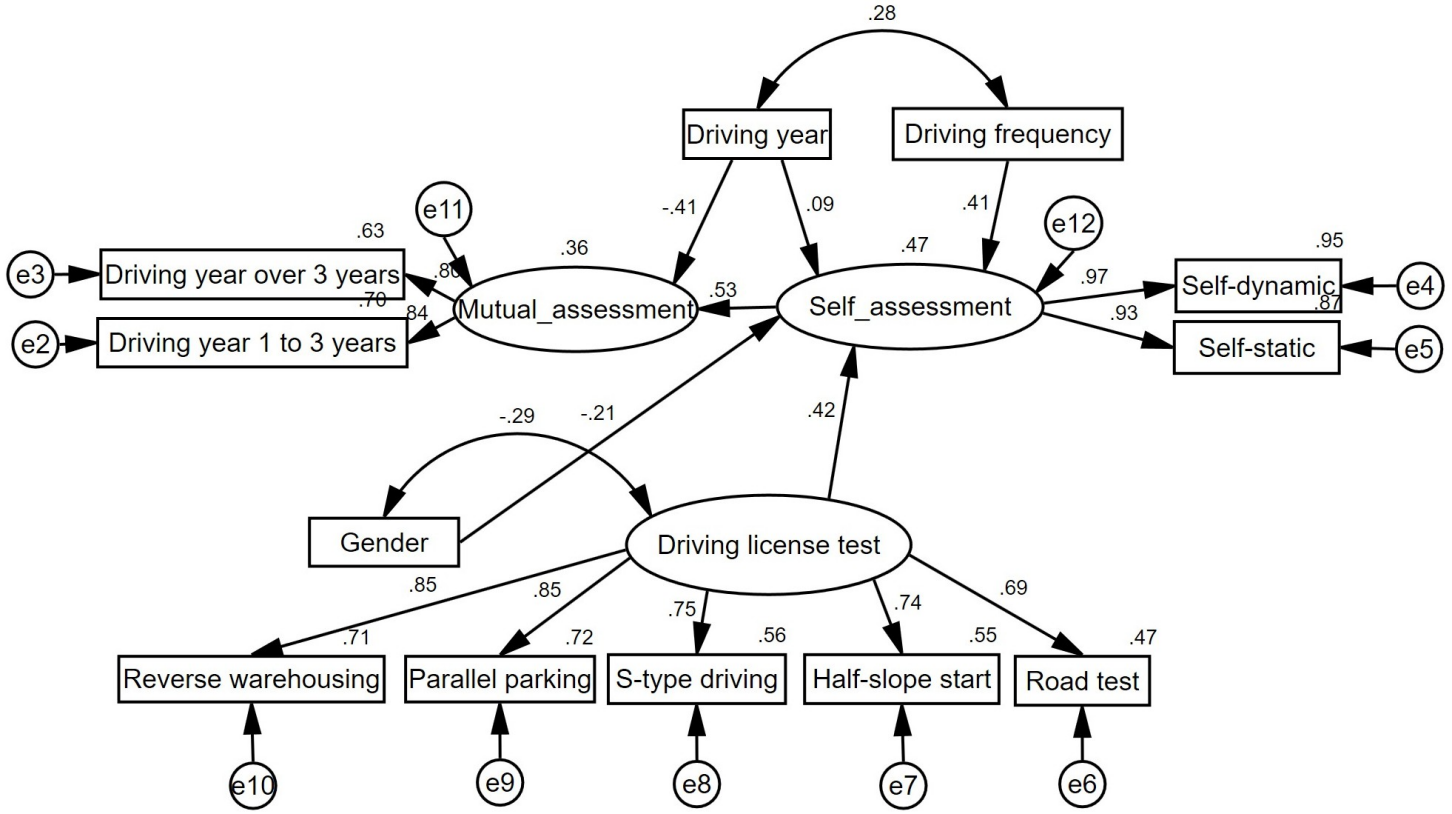
Structural equation model (statistically significant at p < 0.05).

Table 8 shows the definitions and input codes used in the model. The basic structural correlations among all the variables are shown in Fig 10, and the effects of the hypothesized latent variables on coping capacities are quantified.

**Table 8:** Description of model variables.

**Latent factor**	Coping capacity
**Gender**	Gender (Male = 1, Female = 2)
**Driving years**	Driving years (within 1 year = 1, 1 to 3 years = 2, Over 3 years = 3)
**Driving frequency**	Driving frequency (0 to 2 times = 1, 3 to 5 times = 2, Over 5 times = 3)
**Driving license test**	Reverse warehousing
	Parallel parking
	S-type driving
	Half-slope start
	Road test
**Self-assessment**	Dynamic coping capacity (Interact with other vehicles or pedestrians)
	Static coping capacity (No interaction with other vehicles or pedestrians)
**Mutual assessment**	Comprehensive coping capacity (1 to 3 years)
	Comprehensive coping capacity (Over 3 years)

It shows that drivers’ driving years are positively correlated with drivers’ self-assessment scores (with an effective value of 0.09), and the self-assessment scores of experienced drivers are higher than those of less experienced drivers. However, drivers’ driving years are negatively correlated with drivers’ mutual-assessment scores (with an effective value of -0.41), and the scores provided by experienced drivers are lower than those scores provided by less experienced drivers. The SEM clearly shows that drivers’ driving frequency and driving license tests are positively correlated with drivers’ self-assessment scores (with effect values of 0.41 and 0.42, respectively), and drivers’ genders are negatively correlated with their self-assessment scores (with an effect value of -0.21). Self-assessment scores provided by drivers who drive frequently or perform well in their driving license tests are higher than those scores provided by drivers who drive less frequently or do not perform well in their driving license tests. Moreover, self-assessment scores provided by male drivers are higher than those scores provided by female drivers. The SEM also shows that drivers’ SAS are positively correlated with their MAS (with an effective value of 0.53), and drivers who provide a high self-assessment score tend to provide a high mutual assessment score.

The SEM results mentioned above are consistent with the results provided in the previous sections.

There are some other points that need to be explained. Firstly, the covariate correlation coefficient between gender and driving license test is negative (with a value of -0.29), indicating that female drivers score lower or perform worse than male drivers in driving license tests. This result conforms to the real situation. Secondly, the SEM shows a positive correlation coefficient between driving year and driving frequency (with a value of 0.28), indicating that drivers’ driving frequency increases with their driving years. Our explanation for this finding is that most of the participants in this survey are college students, who mostly do not own a car in the first year after they get their driving licenses. However, when they get older, most of them gain the economic strength to own a car and have the opportunity to drive frequently. In such circumstances, there exists a positive correlation between driving year and driving frequency.

As shown in Table 9, all path coefficients (except for the “driving year—self-assessment” path, which has an insignificant coefficient p-value that is lower than 0.05) have a significant p-value below 0.001, indicating that the model results are significant.

**Table 9:** Results of structural equation model on regression weights and standardized regression weights.

Path			Un-standardized Estimate	S.E.	C.R.	P	Standardized Estimate
Self-assessment	<---	Driving frequency	.604	.066	9.113	***	.410
Self-assessment	<---	Driving license test	.484	.062	7.810	***	.424
Self-assessment	<---	Driving years	.141	.072	1.961	.050	.088
Self-assessment	<---	Gender	-.509	.113	-4.526	***	-.205
Mutual assessment	<---	Self-assessment	.439	.050	8.793	***	.530
Mutual assessment	<---	Driving years	-.539	.076	-7.137	***	-.405
Mutual_1to3years	<---	Mutual assessment	1.000				.837
Mutual_over3years	<---	Mutual assessment	.945	.090	10.487	***	.796
Self-dynamic	<---	Self-assessment	1.000				.975
Self-static	<---	Self-assessment	.975	.034	28.614	***	.930
Road test	<---	Driving license test	1.000				.689
Half-slope start	<---	Driving license test	1.265	.105	12.042	***	.745
S-type driving	<---	Driving license test	1.146	.095	12.106	***	.749
Parallel parking	<---	Driving license test	1.494	.111	13.468	***	.848
Reverse warehousing	<---	Driving license test	1.459	.109	13.434	***	.845

***At 0.001 significance level.

In order to examine the fitness of the data, a wide range of fitting criteria have been considered and are listed in Table 10. It shows that all the fitted variables have met the model requirements, indicating that the model adopted in this study has reached an acceptable level of fitting.

**Table 10:** Fitting degree test results ofstructural equation model.

Fit Index	Acceptable Fit	Model Value
CMIN/DF (Chi-square/*df*)	<3	2.471
RMSEA (Root Mean Square Error of Approximation)	<0.08	0.068
GFI (Goodness of Fit Index)	>0.9	0.941
AGFI (Adjusted Goodness of Fit Index)	>0.9	0.906
CFI (Comparative Fit Index)	>0.9	0.964
NFI (Normed Fit Index)	>0.9	0.941
TLI (Tucker-Lewis Index)	>0.9	0.951
IFI (Incremental fit index)	>0.9	0.964

### Cognitive bias

#### Cognitive biases of drivers in different driving year groups

**Fig 11:**
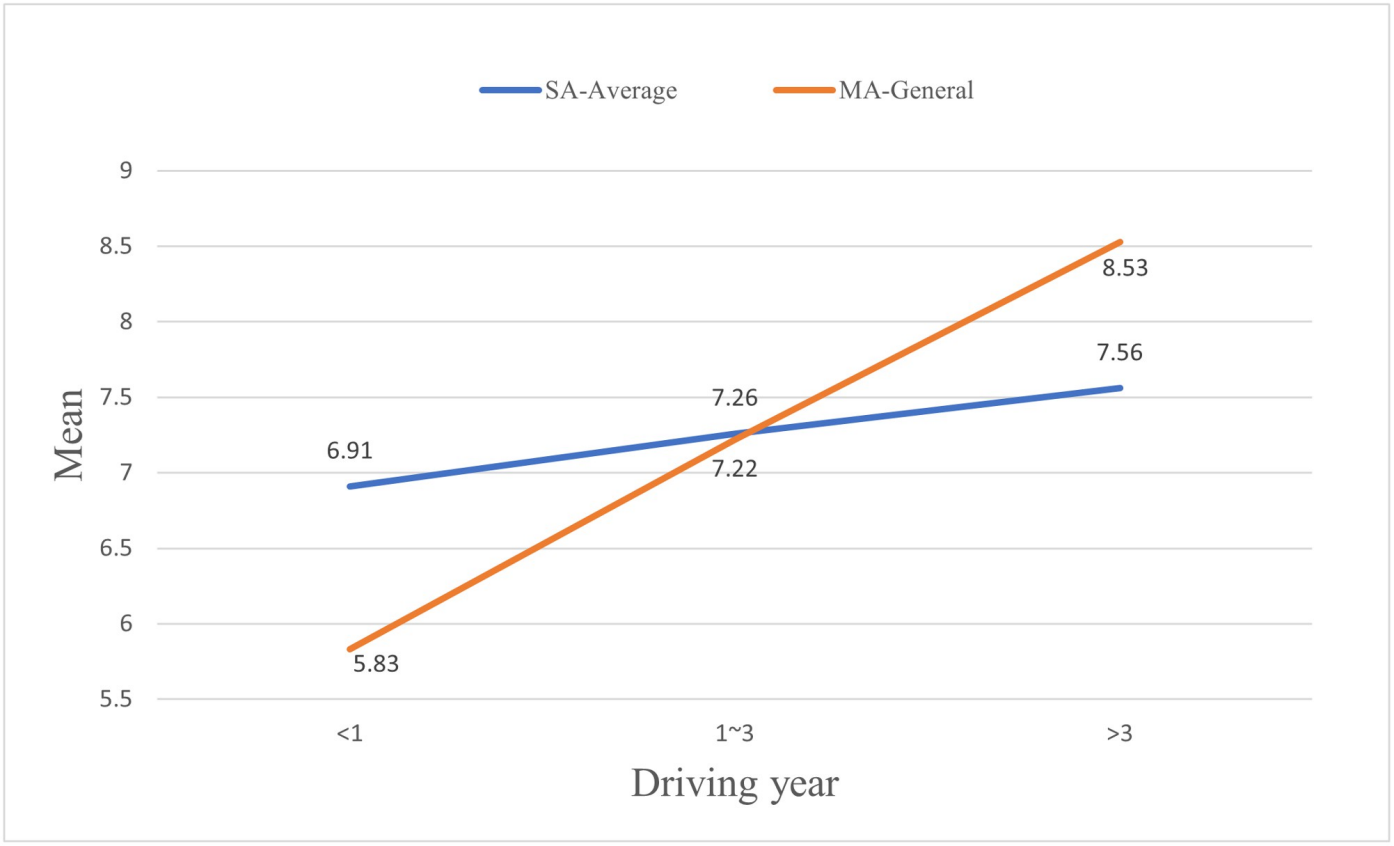
Comparison of mean self-assessment and mutual assessment scores.

To make a clearer comparison with the mutual assessment, a composite self-assessment variable is introduced. The value of the composite self-assessment variable is the average of the dynamic self-assessment scores and the static self-assessment scores. Fig 11 shows the comparison under different driving year conditions. SAS of first-year drivers are much higher than their MAS, and the SAS of drivers with 1–3 years of driving experience are slightly higher than their MAS. However, the SAS of drivers with more than 3 years of driving experience are lower than their MAS.

**Fig 12:**
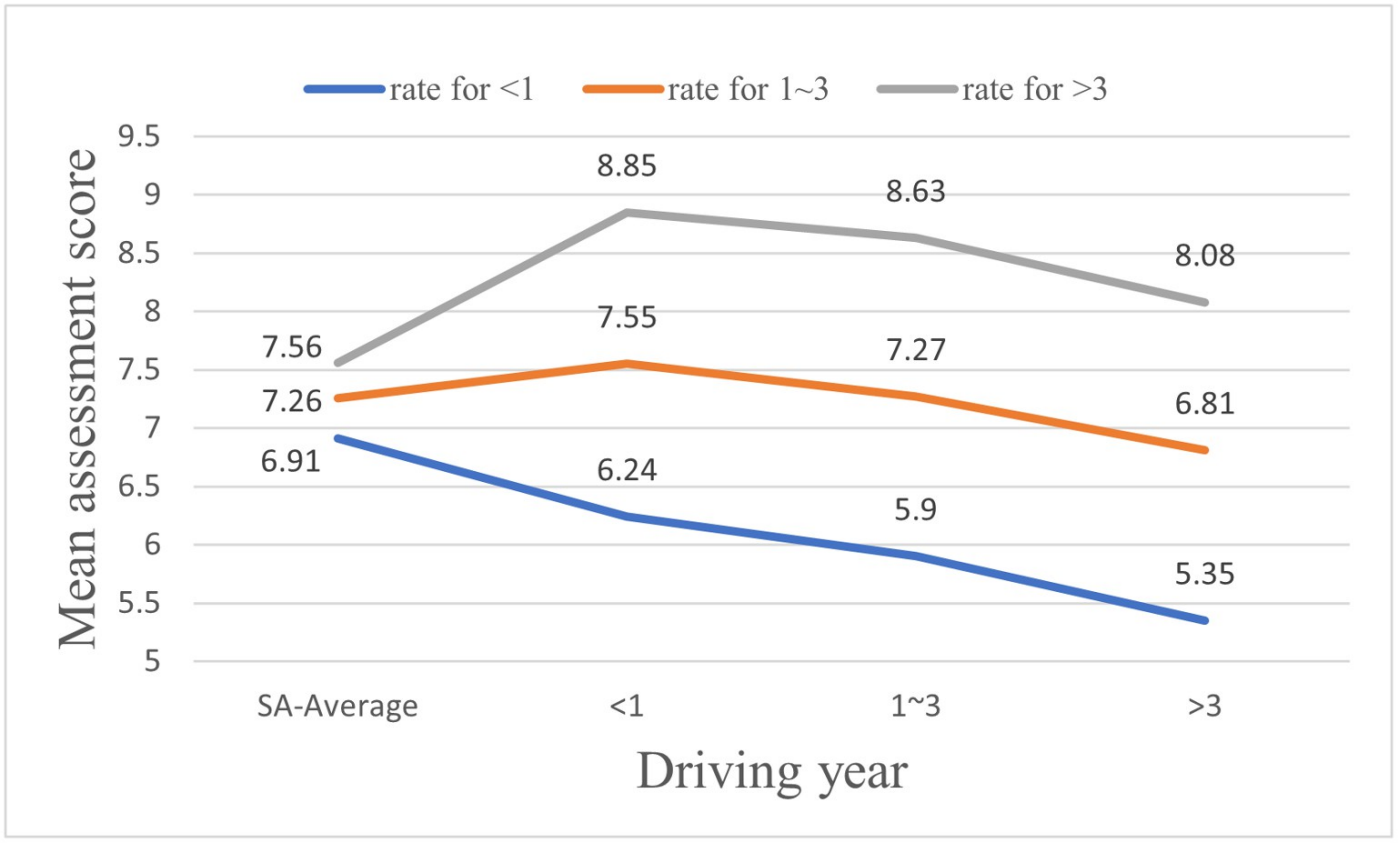
Comparison of self-assessment and mutual assessment scores.

This study has performed a T-test to investigate the differences between drivers’ SAS and MAS so as to reveal drivers’ cognitive biases. Detailed differences between drivers’ SAS and MAS in each driving year group are shown in Table 11 and Fig 12. SAS of first-year drivers are significantly different from the MAS given by drivers of all driving year groups (e.g. “<1”→“<1”, “1~3”→“<1”, “>3”→“<1”). The mean SAS of first-year drivers is 6.91, which is much higher than the mean MAS given by other drivers (i.e., 6.24, 5.9, and 5.35 given by the drivers in the first-year driving group, the 1–3 years driving group, and the over 3-years driving group, respectively). For drivers with 1–3 years of driving experience, there are significant differences between their SAS and MAS given by drivers in the over 3-years driving group, with a T-test result of 2.449 (7.26 vs. 6.81). Different from drivers in the other two groups, drivers in the over 3 years driving group have a mean SAS, which is slightly lower than the mean MAS given by drivers in each of the three driving groups. The mean SAS of drivers in this group is 7.56, while the mean MAS given by drivers in the first-year driving group, the 1–3 years driving group, and the over-3 years driving group are 8.85, 8.63, and 8.08, respectively.

**Table 11:** Differences between drivers’ self-assessments and mutual assessments.

Variables		T-test Result
Self	Mutual	
**<1**	<1→<1	4.495**
	1~3→<1	7.091**
	>3→<1	8.210**
**1~3**	<1→1~3	-1.914
	1~3→1~3	-0.081
	>3→1~3	2.449*
**>3**	<1→>3	-6.427**
	1~3→>3	-5.331**
	>3→>3	-2.360*

**At 0.01 significance level.

*At 0.05 significance level.

This study has also investigated the differences among the mutual assessment scores of all drivers. Drivers assessing other drivers, as well as the drivers assessed by other drivers, were divided into three groups of driving years in this study (e.g. “<1”, “1~3”, “>3”). Mutual assessment scores could be analyzed in two ways (See Table 12 and Fig 12). For drivers in a same driving year group, their MAS given to drivers in different driving year groups vary significantly. Compared with less experienced drivers, experienced drivers provide much higher MAS to themselves and other drivers, as shown in Fig 12. Similarly, for drivers in a same driving year group, their MAS provided by drivers in different driving year groups vary significantly, with the lowest scores coming from drivers in the over-3 years driving group, the medium scores coming from drivers in the 1–3 years driving group, and the highest scores coming from first-year drivers.

**Table 12:** Differences among mutual assessments of drivers in different driving year groups.

Mutual assessment scores		Mutual assessment scores	
provided	Results	received	Results
“<1”→“<1”,“<1”→“1~3”,“<1”→“>3”	1.172**	“<1”→“<1”,“1~3”→“<1”,“>3”→“<1”	10.216**
“1~3”→“<1”,“1~3”→“1~3”,“1~3”→“>3”	173.997**	“<1”→“1~3”,“1~3”→“1~3”,“>3”→“1~3”	9.175**
“>3”→“<1”,“>3”→“1~3”,“>3”→“>3”	78.557**	“<1”→“>3”,“1~3”→“>3”,“>3”→“>3”	10.604**

**At 0.01 significance level.

*At 0.05 significance level.

#### Meta-analysis of cognitive biases

In this section, the results on coping capacities of novice drivers obtained in this study and previous studies are to be compared. Drivers’ individual factors, such as gender, driving year, driving frequency, driving time, etc. can affect drivers’ driving abilities and behaviors, as shown in Table 13.

**Table 13:** Meta-analysis of influencing factors on cognitive biases.

Variable	Reference		This paper
	Result	Author	Result
Gender	Female drivers drive more safely but have fewer capacities of judging, executing, adapting, and mentally coping in their driving.	[76]	Consistent
Driving Years	New drivers are less capable of dealing with such scenarios as slow or backward-moving cars in front of them.	[77]	Consistent
Driving frequency	Drivers driving more frequently have greater coping capacities.	[78]	Consistent

This paper has shown that a driver’s gender, driving years, driving time, and driving frequency all have a significant effect on that driver’s coping capacity under both static and dynamic conditions. Consistent with previous studies, this study has shown that male drivers have a higher coping capacity than female drivers. Generally, experienced drivers (e.g., more driving years or longer driving time) have a larger coping capacity than less experienced drivers.

Table 14 shows the cognitive biases presented by drivers’ self-assessments and mutual assessments. The cognitive bias levels presented in these assessments vary with the evaluation methods on novice drivers (Are they evaluated by an average, normal, or expert observer?) In this study, the proportion of novice drivers who overestimate their coping capacities is lower than that proportion in previous studies. A possible reason is that a different evaluation method is used in this study compared to the previous studies. In this study, novice drivers have been evaluated by all drivers, indicating an average comparison.

**Table 14:** Meta-analysis of cognitive biases presented by drivers’ self-assessments and mutual assessments.

Reference		This paper
Author	Result	Result
[79]	Male drivers are more confident and alert than female drivers.	80.8% of novice drivers are overconfident, and 19.2% of novice drivers are not overconfident, indicating that novice drivers are more likely to overestimate their coping capacities.
[80]	Young male drivers do not assess their driving performance correctly. Also, young male drivers with different driving skills and experience assess their driving performance differently.	
[81]	95% self-assessment score was higher than the mutual assessment score of experts, 5% self-assessment was lower than the mutual assessment of experts	
[82]	75% of the self-assessment scores are provided by confident drivers. 25% of the self-assessment scores are provided by less confident drivers.	

## Discussion

Drivers’ self-assessments show that such driving factors as gender, driving years, driving frequency, and driving time all have a significant effect on drivers’ SAS on coping capacities. Generally, male drivers have a higher SAS than female drivers [83]. And experienced drivers (with more driving years, higher driving frequency, and longer driving time) are more confident than less experienced drivers. All these findings obtained in this study are consistent with the results of previous studies [77,78,84]. This may be due to the fact that females have a conservative attitude towards the assessment of their coping ability due to biological, psychological, and socialization factors. It has been shown that gender is associated with psychological stress, with females being more likely to be stressed than males under the same conditions [85], reacting slower in driving stressful situations, and having poor coping skills for unexpected situations [86]. Physiologically, adult males are on average 10 centimeters taller than females and have greater arm and leg length dimensions than females. Other conditions are the same, the lower the height, the worse the field of vision. Not only is not conducive to observing the road and vehicle conditions, but also more prone to fatigue [87]. Some studies have shown that males have better spatial ability than females. Spatial ability tests three different aspects, namely mental rotation, spatial perception, and spatial visualization [88]. In spatial perception, distance is particularly important for drivers to accurately judge the distance of objects and vehicles to ensure safe driving. Therefore, spatial perception is crucial for drivers’ coping capacity. In Japan, a test was done on the reaction time of male and female drivers, and the test results showed that the braking distance of female drivers was as much as 4 meters longer than that of male drivers. In addition to the difference in reaction ability, the difference in coordination of body movement is also an important reason. Experienced drivers are more confident than novice drivers because they drive longer and more frequently, so they experience more traffic scenarios and have a higher probability of experiencing complex traffic environments [89].

Also, drivers’ mutual assessments show that the driving factors of gender, driving years, and driving frequency all have a significant effect on drivers’ MAS on their coping capacities, which has been verified by previous studies [90–92]. This may be due to the fact that in life, female drivers are subjected to the stereotype that "female drivers are bad at driving", and the existence of female stereotypes stems from the entertainment function of the media, the presupposition of the media on the construction of female images, the gender culture, and the differentiated cognition of male and female on technology. Moreover, people tend to believe that the coping capacity of experienced drivers is higher.

Therefore, driving year showed a positive correlation with the self-assessment of coping capacity and a non-significant relationship with the mutual assessment of coping capacity, and hypothesis (i) is partially supported. Drivers’ self-assessments and mutual assessments vary with their driving years significantly, which is proved by the SEM model described in the previous section.

Cognitive biases are tendencies of people to filter obtained information through their own beliefs and experience when processing information [93]. Differences between drivers’ SAS and MAS, as well as differences between drivers’ MAS received and MAS provided could be regarded as cognitive biases. With the results shown in the previous section, two kinds of cognitive biases have been identified in this study.

The first one is induced by the overconfidence of novice drivers. For first-year drivers, their SAS are much higher than the MAS they receive. In the case that experienced drivers (for example, drivers with more than three years of driving experience) have provided objective mutual-assessment scores, 80.8% of novice drivers, especially those first-year drivers, are overconfident in evaluating their own driving skills. In other words, although first-year drivers agree that their coping capacities are not as great as those capacities of more experienced drivers, they will argue that their driving skills are more outstanding than those skills of other first-year drivers.

If novice drivers are really as skilled and low-risk as they report in the questionnaire, then their "overconfidence" is a kind of "confidence" based on objective facts. However, many scholars have pointed out that the accident rate for novice drivers is much higher than for other groups. Crundall D et al. stated that novice drivers are more likely to be involved in traffic accidents than experienced drivers since novices do not have the same range of observation as experienced drivers when driving a vehicle and that novice drivers observe a smaller range of observation and are therefore more likely to be involved in traffic accidents than experienced drivers [94]. Young novice drivers aged 18–24 have high rates of traffic accidents and road fatalities in high-income countries, middle-income countries and low-income countries [95]. These data suggest that young drivers do not drive as safely as they themselves report and that their estimates of their abilities and their assessments of risk are not as accurate as they might be. This is consistent with previous findings that compared with skilled drivers, less skilled drivers perform poorly in their self-assessments [91]. However, Kong L. found experienced young student drivers tended to overestimate their own driving skills, while the opposite was true for inexperienced young student drivers [53]. This is different from the results of this study, which may be due to the different experimental samples. Kong’s experimental sample was college students who received higher education, whereas this experimental sample did not have a specific identity. And some studies have pointed out that the level of education affects the questionnaire results [96].

The second cognitive bias comes from those more experienced drivers who tend to provide lower mutual assessment scores than those less experienced drivers. As shown in this study, first-year drivers provide the highest mutual assessment scores, and drivers with more than three years of driving experience provide the lowest mutual assessment scores. Therefore, hypothesis (ii) is supported. It can be explained by the more conservative and safer driving behaviors of experienced drivers [97]. Experienced drivers have traveled more miles and hours and encountered more complex scenarios than novice drivers, so coping capacity does improve with the driving experience. Some studies found that in demanding sections, experienced drivers have been found to be more sensitive to the complexity of the road than novice drivers [98–100]. Ma et al. found that experienced drivers responded faster under dangerous circumstances, and experience could help improve drivers’ processing efficiency of danger information [101]. However, they know that driving is not as simple as just driving a car, and that there are many accident-prone points in traffic where you can get involved in an accident if you’re not careful, such as mountainous roads, and mixed traffic scenarios, so they gave a relatively conservative score.

Moreover, cognitive bias may also come from those participants in the survey who want to provide positive answers to the researchers even though their driving skills are below average [102]. Without face-to-face interactions between participants and researchers, the form of an online survey applied in this study could make the situation worse. More likely, drivers with a lack of accurate awareness of their own driving abilities will inaccurately assess their coping capacities [91]. It can well explain the first cognitive bias induced by the overconfidence of novice drivers, as proposed in this study.

First-year drivers who provide the highest mutual assessment scores have positive attitudes towards the coping capacities of other drivers, while drivers with more than three years of driving experience, who provide the lowest mutual assessment scores, have conservative attitudes towards the coping capacities of other drivers. A possible reason is that first-year drivers are the least experienced drivers among all drivers investigated.

Novice drivers usually keep a considerable distance from cars in front of them or drive below a normal speed, allowing them enough time to react and making their daily driving less difficult [103,104]. Therefore, it creates an illusion of safety and control, which makes novice drivers be overconfident with their coping capacities. However, the more they drive, the more complex traffic situations they will encounter. For example, the car in front of a novice driver could suddenly brake in emergency, or a pedestrian could suddenly get in his way from a right-hand parking strip. All these situations will make novice drivers more aware of their limitations in coping capacity.

**Fig 13:**
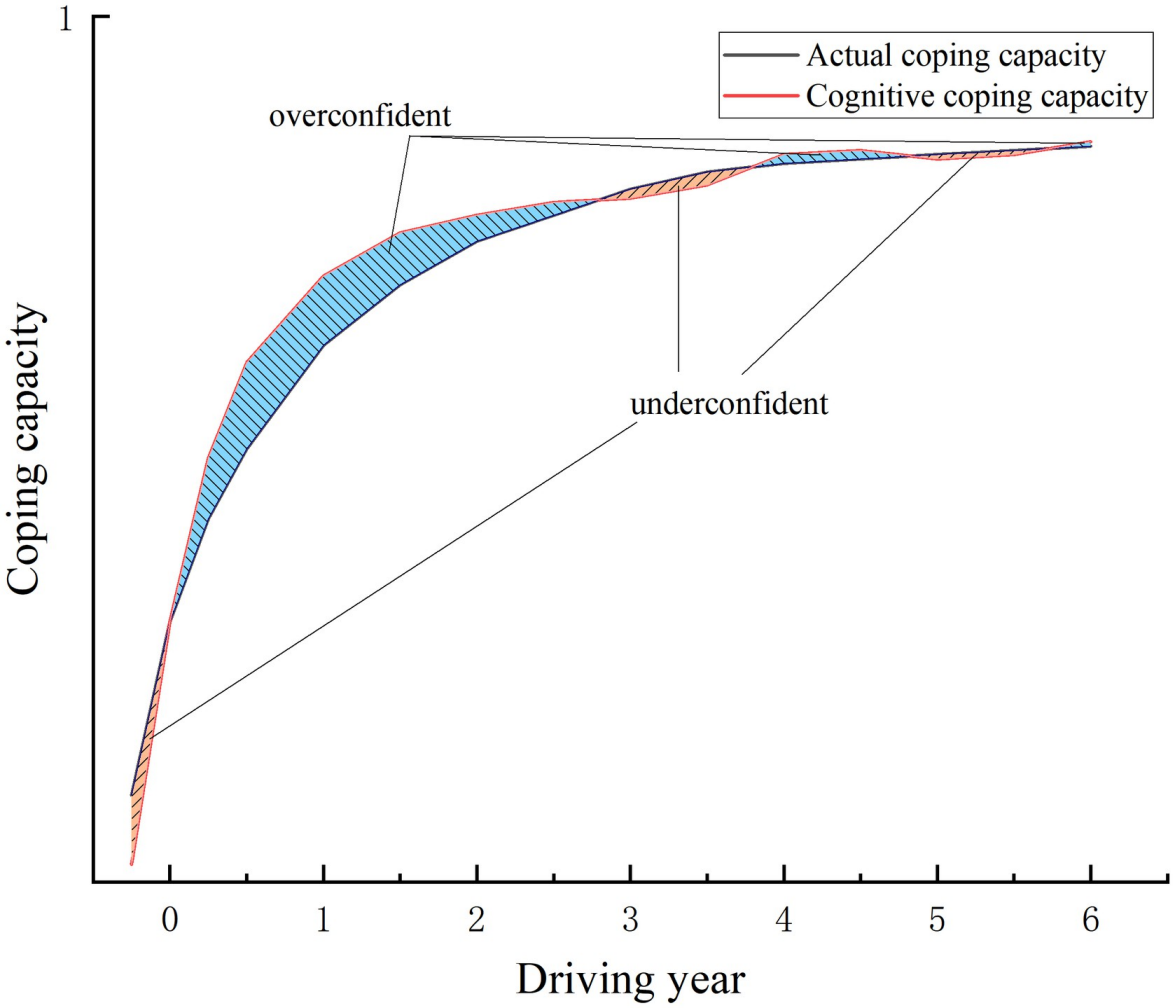
Influencing mechanisms of the driving year factor on the coping capacities of experienced and less-experienced drivers.

Based on the above analysis, we have explored the influencing mechanism of the driving year factor on novice drivers’ coping capacities (See Fig 13). Novice drivers are generally overconfident, especially those first-year drivers. The more regular driving they experience, the more capacities novice drivers will develop to cope with simple traffic situations. When they become more confident, novice drivers will challenge themselves with more difficult driving tasks. For example, they will drive close to the cars in front of them or drive at high speed [105]. Then they will involve themselves in more complex traffic situations which require a greater driving capacity to cope with. When novice drivers can not handle these complex traffic situations well, they will be more aware of their actual coping capacities, thus reducing their cognitive biases. Even some experienced drivers could become less confident after encountering more complex traffic situations, especially those situations that develop into traffic accidents [106]. In such circumstances, even experienced drivers will become less confident. However, with time passing by and driving with no accidents, these experienced drivers will gain confidence. When experienced drivers have enough driving experience, their actual coping capacity and cognitive coping capacity will be consistent.

So, how can novice drivers improve their driving safety? This paper provides the following three solutions for them. The first one is to reduce their cognitive biases on their own coping capacities. It is feasible to provide feedback to drivers on their driving performance so that drivers can assess their driving skills more accurately. In some European countries, drivers will be provided with feedback after training to assess their performance, which is a part of their license system [107]. For example, if drivers perform poorly in training, reproduced driving scenes will be presented to these novice drivers for them to know their weaknesses clearly. The second one is to improve their coping capacities in driving. Road safety training has been shown to be effective in helping drivers better cope with risky or stressful situations and improve decision making for safer behaviors [108,109]. Road safety training can increase driver experience, and enough experience can shift a driver’s information-gathering methods from a long gaze to multi-frequency, helping to reduce the likelihood of hazards [110]. In turn, drivers’ subjective knowledge and perception of traffic rules can influence their behavior on the road [111]. It is feasible to train drivers, especially novice drivers, to cope with complex or even rare driving scenarios [112]. For example, few novice drivers can maneuver their cars out of an emergency collision successfully because, in the real world, few drivers will encounter such an emergent situation. However, with repeated training, novice drivers’ emergency-response performance can be improved. The third solution is that the traffic management department should educate novice drivers extensively. In China, there is a lack of supervision and management of novice drivers. Foreign countries such as the United States, Canada, New Zealand, and Australia manage novice drivers through the Graduated Driver Licensing Systems (GDLS). There are three periods in these licensing systems, which are the fully supervised learning period, the intermediate period of independent driving with restrictions, and the full-permit driving period. These systems are designed to improve the safety of novice drivers by controlling their risks and allowing them to gain more driving experience by driving in an extended, low-risk, and supervised environment [113]. In addition, regular driver training is also critical to driving safety. The number of car crashes encountered by novice drivers trained by Risk Awareness and Perception Training (RAPT) is less than the number of those car crashes encountered by untrained drivers by 23.70% [114]. Novice drivers trained by Virtual Reality-based Risk Awareness and Perception Training Program (V-RAPT) can perceive 86.25% of potential hazards, which are 2.78 times the potential hazards untrained drivers can perceive. Also, the training is effective for experienced drivers [115].

### Limitations

This study has some shortcomings. (i) High percentage of invalid questionnaires. First, this survey was conducted online, which generally has a lower response rate and lower data quality than other survey formats owing to the uncontrollable factors of administration [116–119]. Second, this study is a part of the cognitive bias survey questionnaire and is located at the back of the whole questionnaire, and the evidence of the study suggests that the longer the questionnaire, the lower the response rate [120,121], because the more items a questionnaire has, the lower the motivation to complete it [122]. (ii) This study mainly used a self-reported scale as the research tool. However, survey data are not sufficient for exploring the degrees of influence of these biases. Therefore, the next study will use a driving simulator to test the coping capacities of novice drivers under specific driving scenarios.

## Conclusion

Based on online survey data, this paper has analyzed self-assessment and mutual-assessment scores provided by drivers in different driving year groups. The results show that there are two types of cognitive biases on drivers’ coping capacities. One is induced by the general overconfidence of novice drivers, and the other comes from those more experienced drivers who tend to provide lower mutual assessment scores than those less experienced drivers. Furthermore, in order to present the influences of the driving year factor on drivers’ coping capacities, a chart has been prepared in this study to guide novice drivers to improve their driving safety.

## Supporting information

S1 Appendix Questionnaire. (DOCX)

S1 Dataset All data underlying the findings. (XLSX)
