# Supplementary material for: Analysis of coping capacities and cognitive biases of novice drivers—A questionnaire-based study
Source: PLoS One. 2024 Feb 16;19(2):e0297763. doi: 10.1371/journal.pone.0297763 (PMC10871514; doi:10.1371/journal.pone.0297763)
Supplement: S1 Appendix — (DOCX) [file pone.0297763.s001.docx]

**Investigation on coping capacity of novice drivers**

Dear Sir /Madam：

Hello! We are conducting a survey on novice drivers to accurately analyze the current driving coping capacity for the improvement of novice drivers' driving ability. Your comments are very valuable for our research. We sincerely appreciate your cooperation. Please answer the following questions in order according to your actual feelings. Thank you very much!

1. Do you have a driver's license？

A. Yes B. No

2. The type of your driver's license is？

A. C1（Compact car） B. C2（Automatic car） C. C3（Low-speed truck） D. Else

3. Do you have a car？

A. Yes B. No

4. Your gender is？

A. Man B. Woman

5. How old are you？

A. 18~25 B. 26~40 C. >40 D. Else

6. What is your education background？

A. High school B. Bachelor C. Master D. Else

7. What is your major？

A. Natural science B. Liberal arts C. Else

8. How often do you drive after your driving license obtained？

A. Less than once a week B. One to Two times per week

C. Three to Five times per week D. More than Five times per week

9. How long time of driving for your single travel？

A. Less than 30 minutes B. 30-60 minutes

C. 60-120 minutes D. Longer than 120 minutes

10. Have you ever been involved in a traffic accident？

A. Zero B. One to Two times

C. Three to Five times D. More than Five times

11. Please rate your performance in the driving license test？(score 0-10)

Reverse warehousing

Parallel parking

S-type driving

Half-slope start

Road test

12. If you can coping the dynamic situations (for example, a car ahead suddenly brakes, or someone suddenly rushes out from the right side) timely when driving ? Please fill in score (0-10)

13 If you can coping the static situations (for example, the driver needs to pass through a narrow road, or a running vehicle suddenly skids in a rainy day) timely when driving ? Please fill in score (0-10)

14. Please rate the coping capacity of **other drivers** with different driving experience (score 0-10) :

| Driving experience | Dynamic situations | Static situations |
| --- | --- | --- |
| <1 driving year |  |  |
| 1~3 driving year |  |  |
| >3 driving year |  |  |
